# Awareness of Acquired Hemophilia A among Physicians in Japan: A Web-based Survey

**DOI:** 10.31662/jmaj.2025-0149

**Published:** 2025-09-26

**Authors:** Yoshinobu Seki, Madoka Go, Masahiro Ieko

**Affiliations:** 1 Department of Hematology, Niigata University Medical and Dental Hospital, Niigata, Japan; 2 Department of Hematology, Niigata Cancer Center Hospital, Niigata, Japan; 3 Japan Medical Office, Takeda Pharmaceutical Co., Ltd., Tokyo, Japan; 4 Faculty of Health Sciences, Sapporo University of Health Sciences, Sapporo, Japan

**Keywords:** acquired hemophilia A, awareness, Japan, web-based survey

## Abstract

**Introduction:**

Acquired hemophilia A (AHA) is a rare, life-threatening bleeding disorder caused by the development of autoantibodies against coagulation factor VIII. AHA represents a diagnostic challenge, particularly for non-hematologist physicians who may initially encounter these patients. This study aimed to evaluate the level of AHA awareness among non-hematologist physicians across various medical departments in Japan

**Methods:**

This was a prospective, cross-sectional, observational, web-based survey of non-hematologist physicians, conducted over a period of 2 weeks (April 1-12, 2024) in Japan. The primary endpoint was the participants’ level of AHA awareness.

**Results:**

In total, 4,835 candidate physicians were screened, with 1,701 participants included in the analysis population. Of these, 84.2% had heard of AHA but only 29.7% could identify AHA symptoms and pathologies. More than 45% of participants in the emergency, general medicine, oncology, and rheumatology and collagen medicine departments were familiar with the disease name, symptoms, and pathologies, compared with less than 20% in the geriatrics, neurosurgery, obstetrics and gynecology, orthopedics, respiratory surgery, and urology departments. Although a high proportion (80.4%) of participants reported no experience of examining patients with AHA, many (79.4%) had examined patients exhibiting symptoms of AHA, indicating that they may have encountered patients for whom AHA should have been suspected. When presented with a fictitious AHA case, 44.3% of participants suspected and 34.7% strongly suspected acquired coagulopathy, with substantial variation observed between the different medical departments. Provision of additional laboratory results for the fictitious AHA case increased the proportion of participants who strongly suspected acquired coagulopathy to 38.0%.

**Conclusions:**

Awareness of AHA among non-hematologist physicians in Japan is limited and inconsistent across departments. Educational initiatives are needed to enhance awareness and disease-specific knowledge among physicians, particularly those who are most likely to encounter patients with AHA, which is essential for early diagnosis and treatment.

**UMIN clinical trials registry identification:**

UMIN000053895

## Introduction

Acquired hemophilia A (AHA) is an extremely rare bleeding disorder, with 296 cases documented in Japan in 2022 ^(1)^. AHA is caused by the formation of inhibitory autoantibodies targeting coagulation factor VIII (FVIII) ^(2)^, leading to spontaneous bleeding episodes, such as muscle hematomas, subcutaneous bleeds, and gastrointestinal and urinogenital bleeding ^(3)^. AHA is a serious and life-threatening disease, with mortality in patients with AHA in Japan reported to be 13.8% (8 of 58 patients over 17 months) and 25% (10 of 40 patients over 3 years) in two regional studies ^(4), (5)^.

AHA predominantly affects elderly patients and is associated with an underlying condition in about half of cases ^(6), (7)^. In addition, AHA can be related to pregnancy, with occurrences often reported during the peripartum and postpartum periods ^(7)^. Patients with AHA typically present with abnormal spontaneous bleeding symptoms without a previous bleeding history ^(8)^. Initial investigations for a patient with unexplained bleeding include conducting a full blood count and a coagulation screen ^(3)^. Laboratory tests in patients with AHA typically show a prolonged activated partial thromboplastin time (aPTT), with a normal prothrombin time, along with reduced FVIII activity and the presence of autoantibodies ^(3), (8)^.

Delays in the diagnosis and treatment of AHA may result in poorer patient outcomes and increased mortality ^(9)^. However, patients with AHA often first present to non-hematologist physicians, who may have limited knowledge of blood coagulopathies and lack experience in diagnosing AHA based on the presenting symptoms. Therefore, in some cases, AHA diagnosis is delayed, leading to inadequate treatment and high mortality ^(2), (3)^. To ensure timely diagnosis and treatment of patients with AHA and improve the prognosis of these patients ^(10)^, disease awareness among initial contact departments, including emergency medicine, orthopedics, dermatology, general medicine, and gynecology, is essential. However, data on the awareness of AHA among non-hematologist physicians in Japan are limited.

The aim of this study was to evaluate the awareness of AHA among non-hematologist physicians across various departments in Japan. This survey also aimed to identify the diseases non-hematologist physicians suspect when patients present with spontaneous bleeding and laboratory test results typical of AHA.

## Materials and Methods

### Study design

This was a prospective, cross-sectional, observational, web-based study (UMIN clinical trials registry identification [ID]: UMIN000053895) conducted over a period of 2 weeks in Japan (April 1-12, 2024). Participants completed a web-based survey designed to gather data on their awareness of AHA (Supplementary Table 1). Participants were recruited via an online platform by CareNet, Inc., which also handled consent, registration, and data collection for the study. CareNet, Inc. used central registration numbers to anonymize participants, and only CareNet, Inc. had access to information which would allow the identification of participants. This study was conducted in accordance with the Declaration of Helsinki, the International Society for Pharmacoepidemiology Guidelines for Good Pharmacoepidemiology Practice, the Ethical Guidelines for Medical and Health Research Involving Human Subjects, and applicable local regulations. Participants gave informed consent before answering the survey.

### Participants

Physicians without a hematology specialty were included from the following departments: dermatology, emergency medicine, orthopedics, obstetrics and gynecology, oncology, general medicine, gastroenterology, urology, gastrointestinal surgery, respiratory surgery, neurosurgery, rheumatology and collagen medicine, geriatrics, pediatrics, and general internal medicine. Participants were excluded if, at the time of consent, they had less than 2 years of clinical experience (excluding residency); were working in facilities other than clinics, university hospitals, general hospitals, or national or public hospitals; were not treating patients in an inpatient or outpatient setting; or if legal representatives were required. Participants who either withdrew their consent or failed to answer all the questions within the designated period were considered “dropout” cases and their data were excluded.

### Endpoints

The primary endpoint was the level of AHA awareness, based on participants’ knowledge and understanding of AHA, their experiences with examining patients showing symptoms of AHA, and the number of patients with AHA they had encountered. Secondary endpoints included the proportion of participants who suspected AHA upon encountering a fictional patient with typical bleeding symptoms of AHA.

### Statistical analysis

Owing to the exploratory nature of this study, the sample size was not determined based on statistical calculations. The target number of participants was 1,600 (200 in internal medicine, 100 from each of the other departments; the target number of participants from internal medicine was double that for the other departments because of the large number of internal medicine physicians in Japan), based on feasibility. Summary statistics were calculated for continuous variables. For categorical variables, frequency and proportion (%) were calculated. Participants were categorized by department. Macromill Carenet, Inc. developed the dataset for analyses using QuickCross (original software of Macromill Carenet, Inc.) and statistical analysis was performed by EviPRO Co., Ltd. Any abnormal responses or data requiring exclusion were handled by the primary investigator, with excluded data considered as missing. Imputation of missing values was not conducted.

## Results

### Participant disposition

**Figure 1.**
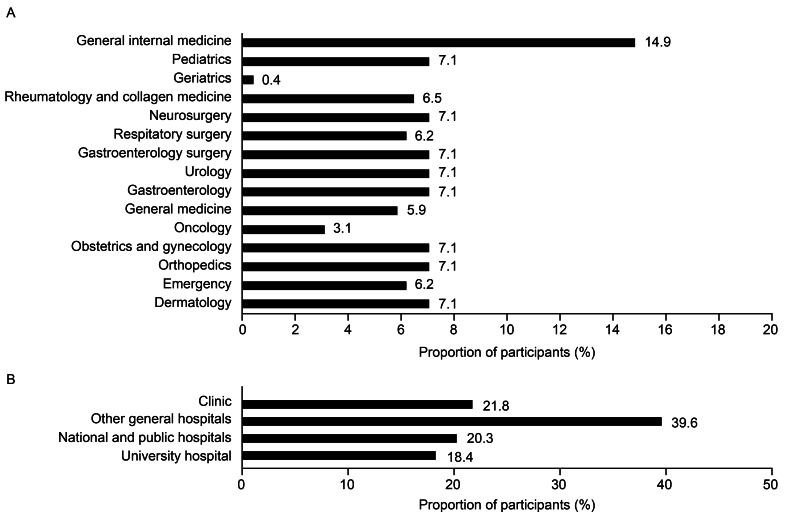
Participants by (A) department and (B) facility (n = 1,701).

In total, 4,835 candidates accessed the dedicated web system. Of these, 3,134 were excluded for the following reasons: refusal to consent (n = 490), deviation from inclusion criteria (n = 84), meeting the exclusion criteria (n = 409), or passing the screening after the target number was achieved (n = 2,151) (Supplementary Figure 1). The remaining 1,701 participants passed the screening, advanced to the survey, and were included in the analysis population. The department with the greatest proportion of participants was general internal medicine (14.9%) (Figure 1A). Participants were mostly spread evenly across other departments (5.9-7.1%), except for geriatrics (0.4%) and oncology (3.1%). The participants were from university hospitals (18.4%), national and public hospitals (20.3%), clinics (21.8%), and other general hospitals (39.6%) (Figure 1B).

### Participants’ level of AHA awareness

**Figure 2.**
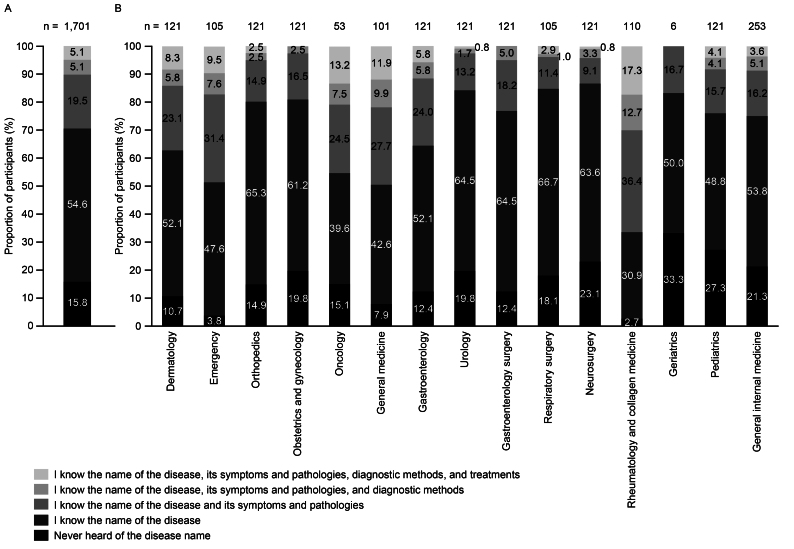
Level of AHA awareness (A) overall and (B) by department (responses to “Select one of the following options that apply to your own knowledge and understanding of ‘acquired hemophilia A’”) (n = 1,701). AHA: acquired hemophilia A.

Among all participants, 84.2% had heard of AHA (Figure 2A). Over half of the participants (54.6%) were only familiar with the disease name, 29.7% could identify symptoms and pathologies, 10.2% understood diagnosis procedures, and 5.1% knew treatments. In the departments of emergency, general medicine, oncology, and rheumatology and collagen medicine, the proportions of participants who knew at least the disease name and its symptoms and pathologies were relatively high (≥45%) compared with other departments (Figure 2B). In the departments of geriatrics, neurosurgery, obstetrics and gynecology, orthopedics, respiratory surgery, and urology, the proportions of participants who were aware of at least the disease name and its symptoms and pathologies were relatively low (<20%) compared with other departments.

### Experience examining patients with AHA

**Figure 3.**
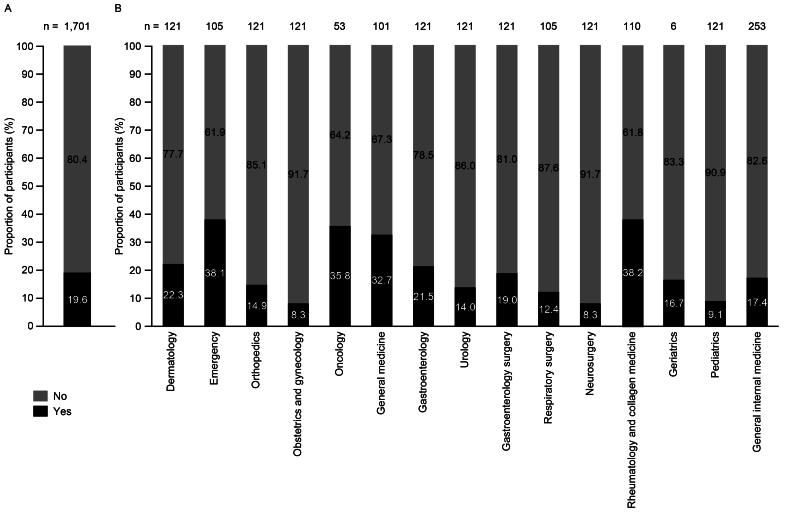
Experience examining patients with AHA (A) overall and (B) by department (responses to: “Have you ever examined patients with ‘acquired hemophilia A’?”) (n = 1,701). AHA: acquired hemophilia A.

Most of the participants (80.4%) reported having no experience examining patients with AHA (Figure 3A). The proportion of participants who had examined patients with AHA was relatively high (>30%) in the emergency, oncology, general medicine, and rheumatology and collagen medicine departments and low (<10%) in the obstetrics and gynecology, neurosurgery, and pediatrics departments (Figure 3B).

### Experience examining patients presenting with AHA symptoms

**Figure 4.**
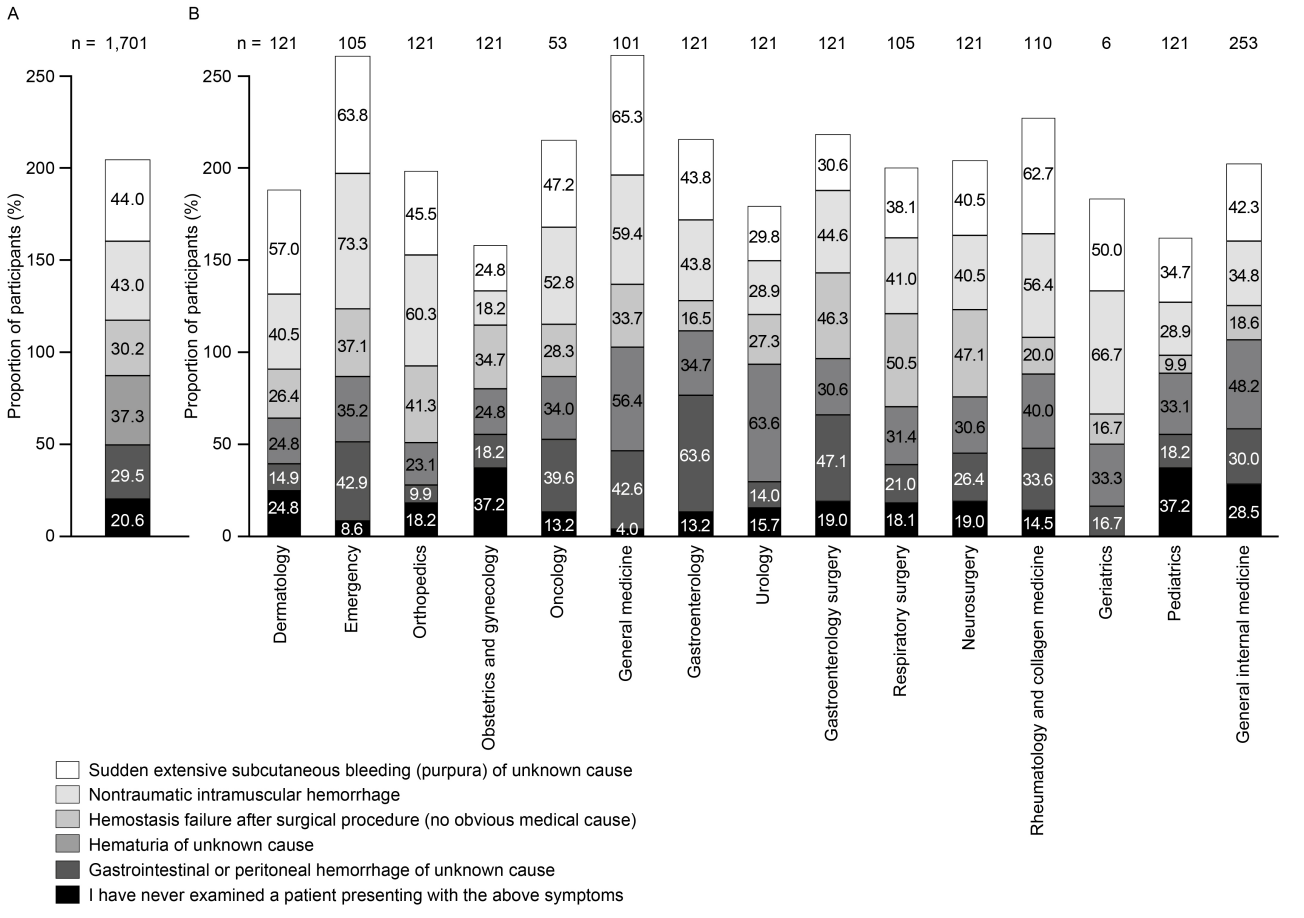
Experience examining patients who present with AHA symptoms (A) overall and (B) by department (responses to: “Select all that apply to your answers from the following symptoms if you have examined patients presenting with...”) (n = 1,701). AHA: acquired hemophilia A.

Among the participants, 44.0% reported experience examining patients with sudden extensive subcutaneous bleeding (purpura) of unknown origin, 43.0% had encountered cases of nontraumatic intramuscular hemorrhage (hematoma), and 37.3% had seen patients with hematuria of unidentified cause (Figure 4A). Compared with other departments, obstetrics and gynecology had the lowest proportion of participants with examination experience of patients with sudden extensive subcutaneous bleeding (purpura) of unknown cause (24.8%) or with nontraumatic intramuscular hemorrhage (hematoma; 18.2%), whereas general medicine had the most experience with sudden extensive subcutaneous bleeding (purpura) of unknown cause (65.3%) and emergency had the most with nontraumatic intramuscular hemorrhage (hematoma; 73.3%) (Figure 4B). The proportions of participants who had examined patients who had sudden extensive subcutaneous bleeding (purpura) of unknown cause from emergency (63.8%), general medicine (65.3%), and rheumatology and collagen medicine (62.7%) departments were higher than for dermatology (57.0%).

### Ability of participants to recognize AHA in a fictional case

**Figure 5.**
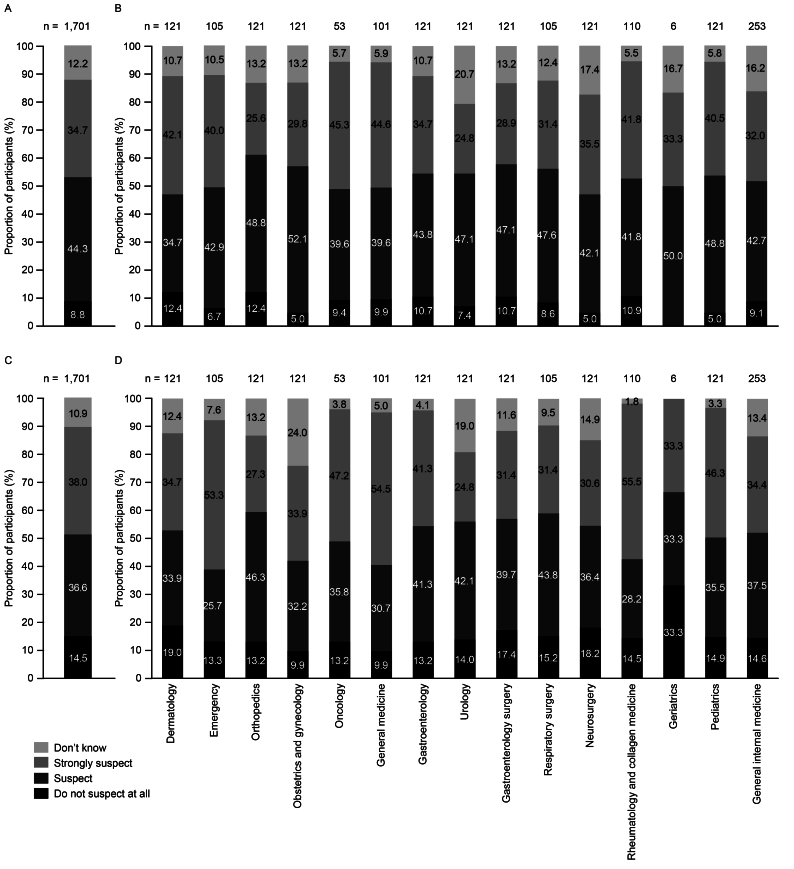
Proportion of participants who suspected an acquired coagulopathy diagnosis based on the fictitious case (A) overall and (B) by department (responses to: fictitious case: “If you were to examine the following patient, which disease would you suspect? Select the level of suspicion for each disease”) and the proportion of participants who suspected an acquired coagulopathy diagnosis when provided with laboratory test results for the fictitious case (C) overall and (D) by department (responses to: fictitious case with clinical laboratory results [Supplementary Table 1]: “For the fictitious case in the previous question, if the following further test results are known, which disease would you suspect? Select the level of suspicion for each disease”) (n = 1,701). AHA: acquired hemophilia A.

**Figure 6.**
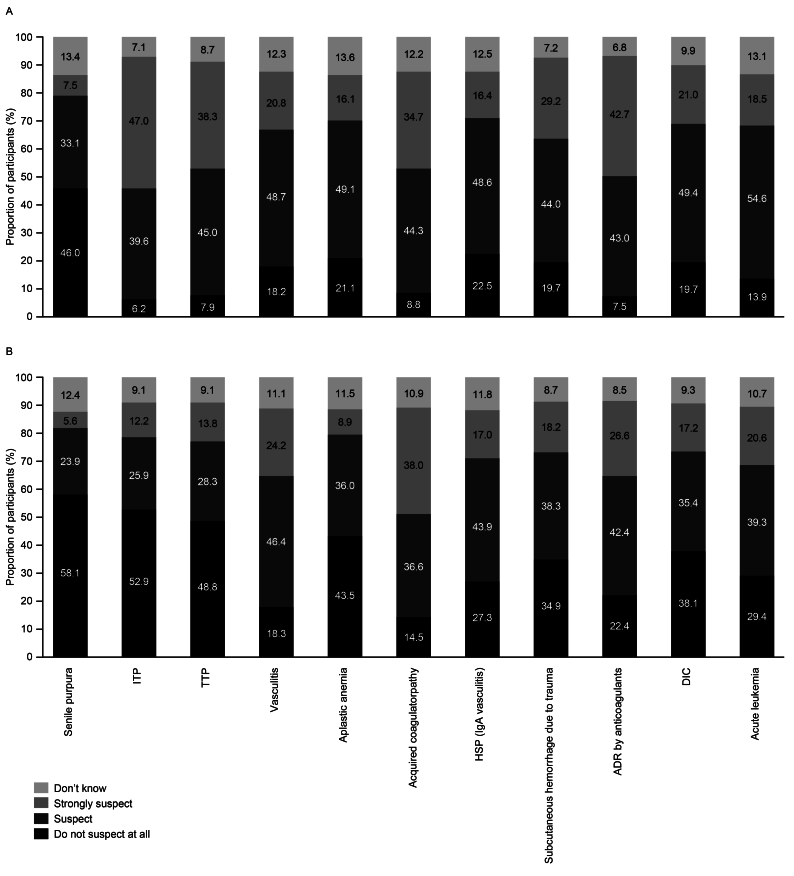
Diseases suspected by participants for the AHA fictional case (A) initially (responses to: fictitious case: “If you were to examine the following patient, which disease would you suspect? Select the level of suspicion for each disease”; 39 participants indicated they suspected or strongly suspected other diseases that were not listed) and (B) when provided with laboratory test results (responses to: fictitious case with clinical laboratory results [Supplementary Table 1]: “For the fictitious case in the previous question, if the following further test results are known, which disease would you suspect? Select the level of suspicion for each disease”; 34 participants indicated they suspected or strongly suspected other diseases that were not listed) (n = 1,701). ADR: adverse drug reaction; AHA: acquired hemophilia A; DIC: disseminated intravascular coagulation; HSP: Henoch–Schönlein purpura; IgA: immunoglobin A; ITP: idiopathic thrombocytopenic purpura; TTP: thrombotic thrombocytopenic purpura.

Participants were given a fictitious case description of a patient displaying characteristic bleeding symptoms of AHA. In total, 44.3% of the participants suspected and 34.7% strongly suspected acquired coagulopathy (Figure 5A). The departments of dermatology, emergency, oncology, general medicine, rheumatology and collagen medicine, and pediatrics had the highest proportions of participants who strongly suspected acquired coagulopathy; however, this proportion remained below 50% across all departments (Figure 5B). Conversely, the proportion of participants who strongly suspected acquired coagulopathy was the lowest in the gastroenterology surgery, orthopedics, obstetrics and gynecology, and urology departments. Some of the other diseases participants strongly suspected were idiopathic thrombocytopenic purpura (47.0%), thrombotic thrombocytopenic purpura (38.3%), and adverse drug reaction by anticoagulants (42.7%) (Figure 6A, Supplementary Table 2).

Upon receiving laboratory test results for the fictitious patient with AHA (including elevated white blood cell counts and C-reactive protein [CRP] levels and prolonged aPTT), the proportion of participants who strongly suspected acquired coagulopathy increased slightly to 38.0% (Figure 5C). However, the proportion of those who suspected acquired coagulopathy decreased slightly to 36.6%, and the proportion of participants who did not suspect acquired coagulopathy at all increased from 8.8% to 14.5%. For emergency, general medicine, and rheumatology and collagen medicine departments, the proportion of participants who strongly suspected acquired coagulopathy increased to more than 50% (Figure 5D). The proportion of participants who strongly suspected acquired coagulopathy decreased in the dermatology and neurosurgery departments. The proportion of participants in the department of geriatrics who did not suspect acquired coagulopathy at all increased from 0% to 33.3% after receiving the test results. Moreover, the proportion of participants who selected “Don’t know” in the obstetrics and gynecology department increased from 13.2% to 24.0%. After receiving the fictitious test results, there were slight increases in the proportion of participants who strongly suspected vasculitis (20.8% to 24.2%), Henoch-Schönlein purpura (16.4% to 17.0%), and acute leukemia (18.5% to 20.6%) (Figure 6B, Supplementary Table 3).

## Discussion

This study assessed the awareness of AHA among 1,701 non-hematologist physicians in Japan. The findings of the survey revealed variance in the awareness of AHA across different medical departments. Although the name of the disease was recognized by most participants (>80%), there appeared to be a gap in the understanding of the presenting symptoms of patients with AHA and the diagnostic process.

Just over 50% of participants only recognized the name “acquired hemophilia A” but did not know more about the disease, suggesting that a substantial portion of physicians from non-hematology departments in Japan lack awareness of AHA symptoms and pathologies, diagnostic procedures, and treatments. Moreover, approximately 16% of participants had never heard of the disease. Participants from departments such as emergency, general medicine, and rheumatology and collagen medicine demonstrated greater knowledge of AHA than other departments, including AHA symptoms and pathologies. This could potentially be attributed to the correlation between AHA and other autoimmune diseases and the acute nature of AHA, with initial symptoms that can be difficult to differentiate from other conditions. Awareness of AHA was lowest in departments focusing on other specialty areas, including geriatrics, neurosurgery, obstetrics and gynecology, orthopedics, respiratory surgery, and urology departments. Geriatrics had the highest proportion of participants who had never heard of the disease name. These findings are particularly concerning given the typical demographic profile of patients with AHA, including elderly individuals and postpartum women ^(7), (11)^, who would usually be treated in these departments.

When presented with a fictitious case of a patient with AHA with common bleeding symptoms, 44.3% of participants suspected and 34.7% strongly suspected a diagnosis of acquired coagulopathy. The proportion of participants who strongly suspected a correct acquired coagulopathy diagnosis for the fictitious case was highest in the dermatology, emergency, general medicine, oncology, pediatrics, and rheumatology and collagen medicine departments. In contrast, the proportion of physicians who strongly suspected an acquired coagulopathy diagnosis was lowest in the orthopedics, obstetrics and gynecology, gastroenterology surgery, and urology departments. This suggests the need for outreach and educational efforts to improve the awareness of AHA and its symptoms across these departments, so that AHA is considered in the differential diagnosis of subcutaneous or intramuscular bleeding symptoms. Some of the other diseases participants strongly suspected for the fictitious case were idiopathic thrombocytopenic purpura (ITP), thrombotic thrombocytopenic purpura (TTP), and adverse drug reaction by anticoagulants. ITP and TTP are diseases that occur more frequently than AHA ^(12), (13), (14)^, and AHA predominantly affects elderly patients who may be taking antiplatelet agents or anticoagulants ^(8)^. Therefore, it is logical that physicians may consider these conditions first when they encounter patients with purpura and muscular bleeding.

When given laboratory test results for the fictitious case, the participants’ opinions shifted, with the proportion of participants who strongly suspected an acquired coagulopathy diagnosis increasing to 38.0%. The highest proportion of participants who strongly suspected a diagnosis of acquired coagulopathy was in the emergency, general medicine, and rheumatology and collagen medicine departments. However, the overall proportion of participants who did not suspect acquired coagulopathy at all also increased once the additional laboratory test results were provided. The laboratory data included elevated white blood cell counts and CRP levels, which may have led some participants to suspect a diagnosis of vasculitis or leukemia. Musculoskeletal bleeding is common in patients with AHA and some patients may also have other autoimmune diseases, such as rheumatoid arthritis ^(7)^, which could contribute to an increase in white blood cells and elevated CRP levels and influence the physicians’ diagnoses.

In addition, the patient’s prolonged aPTT may have been missed by some participants. The questionnaire in this study showed an aPTT test result of 79.6 seconds for the fictious case, which is considered substantially prolonged (with the upper bound of the normal range typically 35-40 seconds) ^(15)^. Despite being provided with this information, a higher proportion of participants suspected leukemia or vasculitis rather than an acquired coagulation disorder, indicating that some physicians may overlook prolonged aPTT results in daily practice. These findings highlight the need to enhance awareness of AHA-related diagnostic tests, particularly, aPTT, among healthcare professionals in Japan.

Despite AHA being considered a very rare disease, about 20% of the surveyed participants had experience examining patients with AHA. More physicians in the emergency, oncology, general medicine, and rheumatology and collagen medicine departments reported experience of examining patients with AHA than in the other departments. The high proportion of participants with experience of examining patients with AHA in general medicine could stem from difficulty in accurately assigning patients to the correct department based only on their initial, often non-specific, symptoms. Approximately 60% of participants from general medicine, dermatology, emergency, and rheumatology and collagen medicine departments had experience examining patients with bleeding symptoms typical of AHA (sudden extensive subcutaneous bleeding [purpura] of unknown cause). More than half of participants from emergency, orthopedics, general medicine, rheumatology and collagen medicine, geriatrics, and oncology departments had encountered patients with nontraumatic intramuscular hemorrhage. Overall, these data suggest that many of the participants had encountered patients for whom AHA should have been suspected.

Data from the European Acquired Hemophilia Registry indicate that delays in diagnosing AHA can substantially affect the duration between the onset of bleeding and the initiation of hemostatic therapy ^(7)^. To effectively manage severe or life-threatening bleeding in patients with AHA, it is essential to increase awareness of the disease among non-hematologist physicians, who often serve as the initial point of contact for these patients. Although our study identified medical specialties with limited awareness of AHA, it did not directly investigate the underlying reasons for these differences. Several factors may contribute to low awareness, including limited clinical exposure to AHA, insufficient training on rare bleeding disorders, and the perception that AHA is not relevant to certain specialties. Furthermore, awareness may also vary based on the severity of bleeding symptoms encountered in clinical practice and the physicians’ experience with bleeding or autoimmune disorders. Future research should investigate these factors to guide targeted education.

Awareness of AHA has also been studied in other countries. In a survey of American physicians conducted in 2010, over 85% of physicians in emergency medicine and critical care identified an underlying bleeding disorder in a case study ^(16)^. However, the survey indicated that emergency medicine and critical care specialists were often hesitant to attribute a patient’s clinical presentation primarily to a bleeding disorder such as AHA and that there was a general lack of appropriate consideration and response to patients’ prolonged aPTT results ^(16)^. Increased collaboration and interaction between hematologists and non-hematologist physicians could be important for bridging this information gap and should be investigated.

Several strategies could be considered to help improve awareness of AHA and support a more accurate and timely diagnosis. For example, the development of simplified diagnostic protocols may help guide physicians when encountering unexplained bleeding. An alert system could be incorporated into electronic health records, so that when prolonged aPTT and subcutaneous bleeding are documented, a prompt is triggered to remind physicians to consider AHA in the differential diagnosis. Moreover, educational interventions and regular dissemination of information regarding AHA across all medical departments may help reinforce awareness. Targeted training sessions, case-based learning, and inclusion of AHA in continuing medical education programs could further support early recognition. It is also worth noting that a separate study (UMIN clinical trials registry ID: UMIN000053897) has investigated diagnostic delays and barriers to AHA diagnosis in Japan, with the findings of that study to be reported separately.

The limitations of the present study include its voluntary and web-based nature, with participants recruited on the CareNet, Inc. platform. This may have introduced selection biases that could affect the generalizability of our findings, including self-selection bias. The survey’s recruitment title of “Survey on Rare Diseases” may have disproportionately attracted participants with clinical interests or experience in rare diseases, potentially leading to an overestimation of overall awareness levels. Moreover, the degree to which participants fully understood the survey questions is unknown and there was potential for recall bias. The participants were not prohibited from consulting textbooks or other sources while completing the survey; therefore, this possibility cannot be excluded. This study did not collect detailed demographic data; therefore, participant age and specific years of clinical experience are not available. In addition, although data on facility types were collected and reported, we did not perform a stratified analysis by institution type because of the limitations in the study design. Furthermore, geographic information (e.g., urban vs rural location) was not obtained. As a result, regional variation in AHA awareness could not be assessed. Finally, given that the survey was conducted solely in Japan, the findings may not be fully applicable to AHA awareness or clinical practice settings in other countries.

In conclusion, awareness of AHA among non-hematologist physicians in Japan is limited and inconsistent across specialties. This indicates a critical need for enhanced educational initiatives to raise disease awareness, which is essential for facilitating the early diagnosis and treatment of patients with AHA and improving their prognosis ^(10)^.

## Article Information

### Acknowledgments

The authors would like to thank all study participants and contributors inside Takeda for their cooperation in conducting the study and preparing the manuscript. Data collection and management was provided by Macromill Carenet, Inc., and statistical analysis was provided by EviPRO Co., Ltd. Medical writing support was provided by Ruby Oberin, PhD and Sarah Graham, PhD of Oxford PharmaGenesis, Melbourne, Australia and funded by Takeda Pharmaceutical Company Limited in accordance with Good Publication Practice (GPP 2022) guidelines (www.ismpp.org/gpp-2022).

### Author Contributions

Yoshinobu Seki, Madoka Go, and Masahiro Ieko contributed to the protocol development and data interpretation. All authors had full access to the data, were actively involved in critically reviewing the manuscript drafts, and approved the final version.

### Conflicts of Interest

This work was supported by Takeda Pharmaceutical Co. Ltd.

Yoshinobu Seki reports grants from Chugai Pharmaceutical, Kyowa-Kirin, and Takeda Pharmaceuticals; contracts for clinical trials from Chugai Pharmaceutical and Takeda Pharmaceuticals; consulting fees from Takeda Pharmaceuticals; and payment/honoraria from Asahi Kasei, Chugai Pharmaceutical, CSL Behring, Novo Nordisk, Sanofi, and Takeda Pharmaceuticals. Madoka Go is an employee of Takeda Pharmaceutical Co., Ltd. Masahiro Ieko reports payment/honoraria from Bayer, Takeda Pharmaceuticals, and Nippon Boehringer Ingelheim. 

### Institutional review board approval code and name of the institution

This study was approved by the Japan Physicians Association Institutional Review Board (management number: 044-2402-01).

### Data Availability Statement

The anonymized data supporting the results presented in this article are available from the corresponding author on reasonable request.

## Supplement

Supplementary Material
