# Supplementary material for: Awareness of Acquired Hemophilia A among Physicians in Japan: A Web-based Survey
Source: JMA J. 2025 Sep 26;8(4):1320–30. doi: 10.31662/jmaj.2025-0149 (PMC12598209; doi:10.31662/jmaj.2025-0149)
Supplement: Supplementary Material [file 2433-3298-8-4-1320-s001.pdf]

Awareness of Acquired Hemophilia A among Physicians in Japan: A Web-Based Survey

Supplementary information

Supplementary Table 1. Questions and Response Options.

Question	Answer options
Select the department at the facility where you are currently work (one answer).	1. Dermatology
	2. Emergency
	3. Orthopedics
	4. Obstetrics and gynecology
	5. Oncology
	6. General medicine
	7. Gastroenterology
	8. Urology
	9. Gastroenterology surgery
	10. Respiratory surgery
	11. Neurosurgery
	12. Rheumatology and collagen medicine

	13. Geriatrics	
	14. Pediatrics	
	15. General internal medicine	
	16. Other clinical department	
Select the management type of the facility where you currently work primarily (one answer).	1. University hospital	
	2. National and public hospitals	
	3. Other general hospitals	
	4. Clinic	
	5. Other facilities	
If you were to examine the following patient, which disease would you suspect? Select the level of suspicion for each disease (one answer each).	Senile purpura	1. Do not suspect at all<br>2. Suspect<br>3. Strongly suspect<br>4. Don’t know
	Idiopathic thrombocytopenic purpura	1. Do not suspect at all<br>2. Suspect<br>3. Strongly suspect

<table>
<tr><td rowspan="5">Fictitious case An 82-year-old man.
Medical history: Rheumatoid arthritis
Family history: Nothing special
This patient came to a hospital complaining that he had been experiencing pain in the left thigh and purpura in the right upper limb without any particular trigger for two weeks. Because there was no worsening of symptoms, the patient was kept under observation without any treatment. Approximately one month later, he visited the hospital again because he felt lightheaded. Pain in the left thigh continued. Extensive purpura expansion was observed mainly on the abdomen and back.
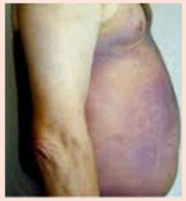 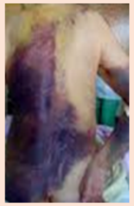
Images from: Collins P, Baudo F, Huth-Kühne A, et al. Consensus recommendations for the diagnosis and treatment of acquired hemophilia A. BMC Res Notes. 2010;3:161.</td><td></td><td>4. Don’t know</td></tr>
<tr><td>Thrombotic thrombocytopenic purpura</td><td>1. Do not suspect at all
2. Suspect
3. Strongly suspect
4. Don’t know</td></tr>
<tr><td>Vasculitis</td><td>1. Do not suspect at all
2. Suspect
3. Strongly suspect
4. Don’t know</td></tr>
<tr><td>Aplastic anemia</td><td>1. Do not suspect at all
2. Suspect
3. Strongly suspect
4. Don’t know</td></tr>
<tr><td>Acquired coagulopathy</td><td>1. Do not suspect at all
2. Suspect</td></tr>
</table>

		3. Strongly suspect<br>4. Don’t know
	Henoch-Schönlein purpura (IgA vasculitis)	1. Do not suspect at all<br>2. Suspect<br>3. Strongly suspect<br>4. Don’t know
	Subcutaneous hemorrhage due to trauma	1. Do not suspect at all<br>2. Suspect<br>3. Strongly suspect<br>4. Don’t know
	Adverse drug reaction by anticoagulants	1. Do not suspect at all<br>2. Suspect<br>3. Strongly suspect<br>4. Don’t know
	DIC	1. Do not suspect at all<br>2. Suspect

		3. Strongly suspect<br>4. Don’t know
	Acute leukemia	1. Do not suspect at all<br>2. Suspect<br>3. Strongly suspect<br>4. Don’t know
	Other (FA)	1. –<br>2. Suspect<br>3. Strongly suspect<br>4. –
For the fictitious case in the previous question, if the following further test results are known, which disease would you suspect? Select the level of suspicion for each disease (one answer each).	Senile purpura	1. Do not suspect at all<br>2. Suspect<br>3. Strongly suspect<br>4. Don’t know

WBC: 18,000/µL[a]	GOT: 38 U/L	BUN: 18.2 mg/dL	PTINR: 1.02
RBC: 314×10^4/µL	GPT: 80 U/L	Cr: 0.36 mg/dL	aPTT: 79.6 s
Hb: 9.7 g/dL	LDH: 518 U/L	Na: 135 mEq/L	FIB: 492 mg/dL
Ht: 28.3%	CPK: 48I U/L	K: 4.4 mEq/L	Ferritin: 292 ng/mL
PLT: 25.1×10^4/µL	TP: 5.5 g/dL	Cl: 100 mEq/L	TIBC: 150 µg/dL
CRP: 11.2 mg/dL	Fe: 23 µg/dL		

[a]No abnormal leukocytes, no abnormalities except neutrophils.

Idiopathic thrombocytopenic purpura	1. Do not suspect at all<br>2. Suspect<br>3. Strongly suspect<br>4. Don't know
Thrombotic thrombocytopenic purpura	1. Do not suspect at all<br>2. Suspect<br>3. Strongly suspect<br>4. Don't know
Vasculitis	1. Do not suspect at all<br>2. Suspect<br>3. Strongly suspect<br>4. Don't know
Aplastic anemia	1. Do not suspect at all<br>2. Suspect<br>3. Strongly suspect<br>4. Don't know

Fictitious case An 82-year-old man.<br>Medical history: Rheumatoid arthritis<br>Family history: Nothing special<br>This patient came to a hospital complaining that he had been experiencing pain in the left thigh and purpura in the right upper limb without any particular trigger for two weeks. Because there was no worsening of symptoms, the patient was kept under observation without any treatment. Approximately one month later, he visited the hospital again because he felt lightheaded. Pain in the left thigh continued. Extensive purpura expansion was observed mainly on the abdomen and back.<br>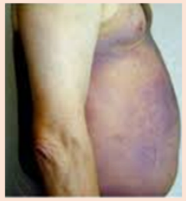 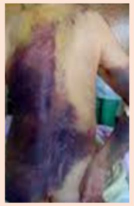<br>Images from: Collins P, Baudo F, Huth-Kühne A, et al. Consensus recommendations for the diagnosis and treatment of acquired hemophilia A. BMC Res Notes. 2010;3:161.	Acquired coagulopathy	1. Do not suspect at all<br>2. Suspect<br>3. Strongly suspect<br>4. Don't know
	Henoch-Schönlein purpur (IgA vasculitis)	1. Do not suspect at all<br>2. Suspect<br>3. Strongly suspect<br>4. Don't know
	Subcutaneous hemorrhage due to trauma	1. Do not suspect at all<br>2. Suspect<br>3. Strongly suspect<br>4. Don't know
	Adverse drug reaction by anticoagulants	1. Do not suspect at all<br>2. Suspect<br>3. Strongly suspect<br>4. Don't know

	DIC	1. Do not suspect at all<br>2. Suspect<br>3. Strongly suspect<br>4. Don’t know
	Acute leukemia	1. Do not suspect at all<br>2. Suspect<br>3. Strongly suspect<br>4. Don’t know
	Other (FA)	1. –<br>2. Suspect<br>3. Strongly suspect<br>4. –
Select one of the following options that apply to your own knowledge and understanding of ‘acquired hemophilia A’ (one answer).	1. Never heard of the disease name<br>2. I know the name of the disease<br>3. I know not only the name of the disease but also its symptoms and pathologies	

	4. I know not only the name of the disease, its symptoms and pathologies but also diagnostic procedures<br>5. I know not only the name of the disease, its symptoms and pathologies, diagnostic methods but also treatments
Select all that apply to your answers from the following symptoms if you have examined patients presenting with (multiple answers allowed).	1. Sudden extensive subcutaneous bleeding (purpura) of unknown cause<br>2. Nontraumatic intramuscular hemorrhage (hematoma)<br>3. Hemostasis failure after surgical procedure (no obvious medical cause)<br>4. Hematuria of unknown cause<br>5. Gastrointestinal or peritoneal hemorrhage of unknown cause<br>6. I have never examined a patient presenting with the above symptoms
Have you ever examined patients with ‘acquired hemophilia A’? (one answer).	1. Yes<br>2. No

aPTT, activated partial thromboplastin time; BUN, blood urea nitrogen; Cl, chloride; CPK, creatine phosphokinase; CRP, c-reactive protein; Cr, creatinine; DIC, disseminated intravascular coagulation; FA, free answer; FIB, fibrinogen; Fe, iron; GOT, glutamic-oxaloacetic transaminase; GPT, glutamic-pyruvic transaminase; Hb, hemoglobin; Ht, hematocrit; IgA, immunoglobin A; K, potassium; LDH, lactate dehydrogenase; Na, sodium; PLT, platelet; PTINR, prothrombin time international normalized ratio; RBC, red blood cell; s, seconds; TIBC, total iron-binding capacity; TP, total protein; WBC, white blood cell

Supplementary Table 2. Diseases Suspected by Participants for the AHA Fictional Case (Stratified by Department).

Suspected disease	Level of suspicion	Dermatology		Emergency		Orthopedics		Obstetrics and gynecology		Oncology		General medicine		Gastroenterology	
		n	n (%)	n	n (%)	n	n (%)	n	n (%)	n	n (%)	n	n (%)	n	n (%)
Senile purpura	Do not suspect at all	121	67 (55.4)	105	56 (53.3)	121	52 (43.0)	121	39 (32.2)	53	18 (34.0)	101	44 (43.6)	121	62 (51.2)
	Suspect		37 (30.6)		32 (30.5)		44 (36.4)		45 (37.2)		27 (50.9)		43 (42.6)		33 (27.3)
	Strongly suspect		14 (11.6)		4 (3.8)		12 (9.9)		6 (5.0)		6 (11.3)		9 (8.9)		13 (10.7)
	Don't know		3 (2.5)		13 (12.4)		13 (10.7)		31 (25.6)		2 (3.8)		5 (5.0)		13 (10.7)
Idiopathic thrombocytopenic purpura	Do not suspect at all	121	14 (11.6)	105	7 (6.7)	121	4 (3.3)	121	6 (5.0)	53	1 (1.9)	101	8 (7.9)	121	4 (3.3)
	Suspect		51 (42.1)		44 (41.9)		56 (46.3)		43 (35.5)		21 (39.6)		40 (39.6)		53 (43.8)
	Strongly suspect		51 (42.1)		49 (46.7)		55 (45.5)		57 (47.1)		27 (50.9)		50 (49.5)		60 (49.6)
	Don't know		5 (4.1)		5 (4.8)		6 (5.0)		15 (12.4)		4 (7.5)		3 (3.0)		4 (3.3)
Thrombotic thrombocytopenic purpura	Do not suspect at all	121	19 (15.7)	105	8 (7.6)	121	7 (5.8)	121	3 (2.5)	53	3 (5.7)	101	13 (12.9)	121	9 (7.4)
	Suspect		50 (41.3)		66 (62.9)		55 (45.5)		46 (38.0)		20 (37.7)		49 (48.5)		67 (55.4)
	Strongly suspect		47 (38.8)		25 (23.8)		50 (41.3)		51 (42.1)		26 (49.1)		35 (34.7)		39 (32.2)
	Don't know		5 (4.1)		6 (5.7)		9 (7.4)		21 (17.4)		4 (7.5)		4 (4.0)		6 (5.0)
Vasculitis	Do not suspect at all	121	41 (33.9)	105	22 (21.0)	121	27 (22.3)	121	18 (14.9)	53	12 (22.6)	101	17 (16.8)	121	21 (17.4)
	Suspect		50 (41.3)		53 (50.5)		53 (43.8)		55 (45.5)		21 (39.6)		49 (48.5)		68 (56.2)
	Strongly suspect		25 (20.7)		15 (14.3)		27 (22.3)		21 (17.4)		16 (30.2)		30 (29.7)		25 (20.7)

	Don't know		5 (4.1)		15 (14.3)		14 (11.6)		27 (22.3)		4 (7.5)		5 (5.0)		7 (5.8)
Aplastic anemia	Do not suspect at all	121	26 (21.5)	105	32 (30.5)	121	25 (20.7)	121	13 (10.7)	53	10 (18.9)	101	22 (21.8)	121	26 (21.5)
	Suspect		60 (49.6)		49 (46.7)		63 (52.1)		59 (48.8)		30 (56.6)		52 (51.5)		66 (54.5)
	Strongly suspect		23 (19.0)		14 (13.3)		15 (12.4)		18 (14.9)		9 (17.0)		23 (22.8)		20 (16.5)
	Don't know		12 (9.9)		10 (9.5)		18 (14.9)		31 (25.6)		4 (7.5)		4 (4.0)		9 (7.4)
Acquired coagulopathy	Do not suspect at all	121	15 (12.4)	105	7 (6.7)	121	15 (12.4)	121	6 (5.0)	53	5 (9.4)	101	10 (9.9)	121	13 (10.7)
	Suspect		42 (34.7)		45 (42.9)		59 (48.8)		63 (52.1)		21 (39.6)		40 (39.6)		53 (43.8)
	Strongly suspect		51 (42.1)		42 (40.0)		31 (25.6)		36 (29.8)		24 (45.3)		45 (44.6)		42 (34.7)
	Don't know		13 (10.7)		11 (10.5)		16 (13.2)		16 (13.2)		3 (5.7)		6 (5.9)		13 (10.7)
Henoch–Schönlein purpura: (IgA vasculitis)	Do not suspect at all	121	66 (54.5)	105	29 (27.6)	121	22 (18.2)	121	15 (12.4)	53	8 (15.1)	101	29 (28.7)	121	16 (13.2)
	Suspect		31 (25.6)		46 (43.8)		63 (52.1)		64 (52.9)		30 (56.6)		49 (48.5)		72 (59.5)
	Strongly suspect		14 (11.6)		15 (14.3)		22 (18.2)		17 (14.0)		12 (22.6)		17 (16.8)		25 (20.7)
	Don't know		10 (8.3)		15 (14.3)		14 (11.6)		25 (20.7)		3 (5.7)		6 (5.9)		8 (6.6)
Subcutaneous hemorrhage due to trauma	Do not suspect at all	121	13 (10.7)	105	11 (10.5)	121	22 (18.2)	121	22 (18.2)	53	12 (22.6)	101	10 (9.9)	121	20 (16.5)
	Suspect		55 (45.5)		40 (38.1)		61 (50.4)		60 (49.6)		26 (49.1)		50 (49.5)		53 (43.8)
	Strongly suspect		48 (39.7)		47 (44.8)		34 (28.1)		23 (19.0)		13 (24.5)		38 (37.6)		43 (35.5)
	Don't know		5 (4.1)		7 (6.7)		4 (3.3)		16 (13.2)		2 (3.8)		3 (3.0)		5 (4.1)
Adverse drug	Do not suspect at all	121	8 (6.6)	105	9 (8.6)	121	5 (4.1)	121	11 (9.1)	53	3 (5.7)	101	2 (2.0)	121	10 (8.3)
	Suspect		44 (36.4)		32 (30.5)		54 (44.6)		60 (49.6)		25 (47.2)		42 (41.6)		47 (38.8)

reaction by anticoagulants	Strongly suspect		62 (51.2)		57 (54.3)		58 (47.9)		35 (28.9)		23 (43.4)		55 (54.5)		57 (47.1)
	Don't know		7 (5.8)		7 (6.7)		4 (3.3)		15 (12.4)		2 (3.8)		2 (2.0)		7 (5.8)
DIC	Do not suspect at all	121	18 (14.9)	105	23 (21.9)	121	28 (23.1)	121	19 (15.7)	53	7 (13.2)	101	17 (16.8)	121	22 (18.2)
	Suspect		50 (41.3)		53 (50.5)		64 (52.9)		52 (43.0)		30 (56.6)		60 (59.4)		61 (50.4)
	Strongly suspect		40 (33.1)		24 (22.9)		16 (13.2)		27 (22.3)		13 (24.5)		20 (19.8)		29 (24.0)
	Don't know		13 (10.7)		5 (4.8)		13 (10.7)		23 (19.0)		3 (5.7)		4 (4.0)		9 (7.4)
Acute leukemia	Do not suspect at all	121	15 (12.4)	105	16 (15.2)	121	24 (19.8)	121	8 (6.6)	53	7 (13.2)	101	10 (9.9)	121	15 (12.4)
	Suspect		55 (45.5)		61 (58.1)		59 (48.8)		62 (51.2)		28 (52.8)		61 (60.4)		78 (64.5)
	Strongly suspect		37 (30.6)		14 (13.3)		15 (12.4)		24 (19.8)		13 (24.5)		26 (25.7)		18 (14.9)
	Don't know		14 (11.6)		14 (13.3)		23 (19.0)		27 (22.3)		5 (9.4)		4 (4.0)		10 (8.3)
Other	Suspect	1	0 (0.0)	5	4 (80.0)	4	2 (50.0)	4	4 (100.0)	1	1 (100.0)	4	4 (100.0)	0	0 (-)
	Strongly suspect		1 (100.0)		1 (20.0)		2 (50.0)		0 (0.0)		0 (0.0)		0 (0.0)		0 (-)

AHA, acquired hemophilia A; DIC, disseminated intravascular coagulation; IgA, immunoglobin A

Supplementary Table 2 continued. Diseases Suspected by Participants for the AHA Fictional Case (Stratified by Department)**.**

Suspected disease	Level of suspicion	Urology		Gastroenterology surgery		Respiratory surgery		Neurosurgery		Rheumatology and collagen medicine		Geriatrics		Pediatrics		General internal medicine	
		n	n (%)	n	n (%)	n	n (%)	n	n (%)	n	n (%)	n	n (%)	n	n (%)	n	n (%)
Senile purpura	Do not suspect at all	121	58 (47.9)	121	54 (44.6)	105	47 (44.8)	121	57 (47.1)	110	70 (63.6)	6	1 (16.7)	121	38 (31.4)	253	120 (47.4)
	Suspect		32 (26.4)		44 (36.4)		37 (35.2)		31 (25.6)		26 (23.6)		2 (33.3)		48 (39.7)		82 (32.4)
	Strongly suspect		3 (2.5)		5 (4.1)		11 (10.5)		10 (8.3)		10 (9.1)		2 (33.3)		5 (4.1)		17 (6.7)
	Don't know		28 (23.1)		18 (14.9)		10 (9.5)		23 (19.0)		4 (3.6)		1 (16.7)		30 (24.8)		34 (13.4)
Idiopathic thrombocytopenic purpura	Do not suspect at all	121	1 (0.8)	121	4 (3.3)	105	5 (4.8)	121	3 (2.5)	110	13 (11.8)	6	0 (0.0)	121	20 (16.5)	253	16 (6.3)
	Suspect		40 (33.1)		46 (38.0)		47 (44.8)		37 (30.6)		41 (37.3)		3 (50.0)		60 (49.6)		92 (36.4)
	Strongly suspect		62 (51.2)		61 (50.4)		49 (46.7)		70 (57.9)		55 (50.0)		2 (33.3)		33 (27.3)		119 (47.0)
	Don't know		18 (14.9)		10 (8.3)		4 (3.8)		11 (9.1)		1 (0.9)		1 (16.7)		8 (6.6)		26 (10.3)
Thrombotic thrombocytopenic	Do not suspect at all	121	3 (2.5)	121	7 (5.8)	105	6 (5.7)	121	3 (2.5)	110	18 (16.4)	6	1 (16.7)	121	10 (8.3)	253	25 (9.9)
	Suspect		49 (40.5)		51 (42.1)		52 (49.5)		44 (36.4)		56 (50.9)		3 (50.0)		49 (40.5)		109 (43.1)

purpura	Strongly suspect		47 (38.8)		52 (43.0)		39 (37.1)		60 (49.6)		34 (30.9)		1 (16.7)		54 (44.6)		92 (36.4)
	Don't know		22 (18.2)		11 (9.1)		8 (7.6)		14 (11.6)		2 (1.8)		1 (16.7)		8 (6.6)		27 (10.7)
	Do not suspect at all		15 (12.4)		20 (16.5)		20 (19.0)		18 (14.9)		18 (16.4)		0 (0.0)		14 (11.6)		47 (18.6)
Vasculitis	Suspect	121	61 (50.4)	121	58 (47.9)	105	56 (53.3)	121	60 (49.6)	110	49 (44.5)	6	4 (66.7)	121	63 (52.1)	253	128 (50.6)
	Strongly suspect		19 (15.7)		23 (19.0)		19 (18.1)		17 (14.0)		39 (35.5)		1 (16.7)		32 (26.4)		45 (17.8)
	Don't know		26 (21.5)		20 (16.5)		10 (9.5)		26 (21.5)		4 (3.6)		1 (16.7)		12 (9.9)		33 (13.0)
	Do not suspect at all		21 (17.4)		23 (19.0)		25 (23.8)		24 (19.8)		32 (29.1)		0 (0.0)		19 (15.7)		61 (24.1)
Aplastic anemia	Suspect	121	54 (44.6)	121	53 (43.8)	105	45 (42.9)	121	57 (47.1)	110	53 (48.2)	6	3 (50.0)	121	70 (57.9)	253	122 (48.2)
	Strongly suspect		16 (13.2)		21 (17.4)		15 (14.3)		21 (17.4)		20 (18.2)		1 (16.7)		22 (18.2)		36 (14.2)
	Don't know		30 (24.8)		24 (19.8)		20 (19.0)		19 (15.7)		5 (4.5)		2 (33.3)		10 (8.3)		34 (13.4)
	Do not suspect at all		9 (7.4)		13 (10.7)		9 (8.6)		6 (5.0)		12 (10.9)		0 (0.0)		6 (5.0)		23 (9.1)
Acquired coagulopathy	Suspect	121	57 (47.1)	121	57 (47.1)	105	50 (47.6)	121	51 (42.1)	110	46 (41.8)	6	3 (50.0)	121	59 (48.8)	253	108 (42.7)
	Strongly suspect		30 (24.8)		35 (28.9)		33 (31.4)		43 (35.5)		46 (41.8)		2 (33.3)		49 (40.5)		81 (32.0)
	Don't know		25 (20.7)		16 (13.2)		13 (12.4)		21 (17.4)		6 (5.5)		1 (16.7)		7 (5.8)		41 (16.2)

Henoch–Schönlein purpura: (IgA vasculitis)	Do not suspect at all	121	14 (11.6)	121	19 (15.7)	105	14 (13.3)	121	15 (12.4)	110	38 (34.5)	6	1 (16.7)	121	44 (36.4)	253	53 (20.9)
	Suspect		58 (47.9)		62 (51.2)		59 (56.2)		60 (49.6)		52 (47.3)		1 (16.7)		54 (44.6)		126 (49.8)
	Strongly suspect		24 (19.8)		23 (19.0)		17 (16.2)		18 (14.9)		18 (16.4)		3 (50.0)		11 (9.1)		43 (17.0)
	Don't know		25 (20.7)		17 (14.0)		15 (14.3)		28 (23.1)		2 (1.8)		1 (16.7)		12 (9.9)		31 (12.3)
Subcutaneous hemorrhage due to trauma	Do not suspect at all	121	34 (28.1)	121	26 (21.5)	105	25 (23.8)	121	25 (20.7)	110	20 (18.2)	6	0 (0.0)	121	29 (24.0)	253	66 (26.1)
	Suspect		38 (31.4)		51 (42.1)		45 (42.9)		56 (46.3)		49 (44.5)		3 (50.0)		57 (47.1)		104 (41.1)
	Strongly suspect		28 (23.1)		33 (27.3)		27 (25.7)		32 (26.4)		40 (36.4)		2 (33.3)		29 (24.0)		59 (23.3)
	Don't know		21 (17.4)		11 (9.1)		8 (7.6)		8 (6.6)		1 (0.9)		1 (16.7)		6 (5.0)		24 (9.5)
Adverse drug reaction by anticoagulants	Do not suspect at all	121	12 (9.9)	121	13 (10.7)	105	5 (4.8)	121	11 (9.1)	110	7 (6.4)	6	0 (0.0)	121	6 (5.0)	253	25 (9.9)
	Suspect		51 (42.1)		49 (40.5)		58 (55.2)		54 (44.6)		28 (25.5)		2 (33.3)		75 (62.0)		111 (43.9)
	Strongly suspect		41 (33.9)		47 (38.8)		37 (35.2)		48 (39.7)		72 (65.5)		3 (50.0)		33 (27.3)		98 (38.7)
	Don't know		17 (14.0)		12 (9.9)		5 (4.8)		8 (6.6)		3 (2.7)		1 (16.7)		7 (5.8)		19 (7.5)
DIC	Do not suspect at all	121	20 (16.5)	121	24 (19.8)	105	24 (22.9)	121	25 (20.7)	110	24 (21.8)	6	1 (16.7)	121	24 (19.8)	253	59 (23.3)

	Suspect		52 (43.0)		64 (52.9)		53 (50.5)		50 (41.3)		58 (52.7)		2 (33.3)		62 (51.2)		129 (51.0)
	Strongly suspect		25 (20.7)		24 (19.8)		18 (17.1)		30 (24.8)		27 (24.5)		2 (33.3)		25 (20.7)		37 (14.6)
	Don't know		24 (19.8)		9 (7.4)		10 (9.5)		16 (13.2)		1 (0.9)		1 (16.7)		10 (8.3)		28 (11.1)
Acute leukemia	Do not suspect at all	121	12 (9.9)	121	20 (16.5)	105	13 (12.4)	121	16 (13.2)	110	21 (19.1)	6	0 (0.0)	121	16 (13.2)	253	43 (17.0)
	Suspect		66 (54.5)		58 (47.9)		61 (58.1)		65 (53.7)		69 (62.7)		3 (50.0)		66 (54.5)		136 (53.8)
	Strongly suspect		17 (14.0)		24 (19.8)		13 (12.4)		22 (18.2)		17 (15.5)		2 (33.3)		29 (24.0)		43 (17.0)
	Don't know		26 (21.5)		19 (15.7)		18 (17.1)		18 (14.9)		3 (2.7)		1 (16.7)		10 (8.3)		31 (12.3)
Other	Suspect	1	1 (100.0)	4	3 (75.0)	1	1 (100.0)	2	2 (100.0)	2	0 (0.0)	0	0 (-)	2	2 (100.0)	8	5 (62.5)
	Strongly suspect		0 (0.0)		1 (25.0)		0 (0.0)		0 (0.0)		2 (100.0)		0 (-)		0 (0.0)		3 (37.5)

AHA, acquired hemophilia A; DIC, disseminated intravascular coagulation; IgA, immunoglobin A.

Supplementary Table 3. Diseases Suspected by Participants for the AHA Fictional Case after Receiving Laboratory Test Results (Stratified by Department).

Suspected disease	Level of suspicion	Dermatology		Emergency		Orthopedics		Obstetrics and gynecology		Oncology		General medicine		Gastroenterology	
		n	n (%)	n	n (%)	n	n (%)	n	n (%)	n	n (%)	n	n (%)	n	n (%)
Senile purpura	Do not suspect at all	121	80 (66.1)	105	63 (60.0)	121	61 (50.4)	121	58 (47.9)	53	33 (62.3)	101	57 (56.4)	121	73 (60.3)
	Suspect		25 (20.7)		27 (25.7)		37 (30.6)		22 (18.2)		14 (26.4)		31 (30.7)		30 (24.8)
	Strongly suspect		11 (9.1)		3 (2.9)		5 (4.1)		8 (6.6)		4 (7.5)		7 (6.9)		8 (6.6)
	Don't know		5 (4.1)		12 (11.4)		18 (14.9)		33 (27.3)		2 (3.8)		6 (5.9)		10 (8.3)
Idiopathic thrombocytopenic purpura	Do not suspect at all	121	66 (54.5)	105	58 (55.2)	121	45 (37.2)	121	58 (47.9)	53	33 (62.3)	101	57 (56.4)	121	60 (49.6)
	Suspect		30 (24.8)		26 (24.8)		49 (40.5)		21 (17.4)		13 (24.5)		28 (27.7)		34 (28.1)
	Strongly suspect		17 (14.0)		13 (12.4)		16 (13.2)		17 (14.0)		6 (11.3)		13 (12.9)		22 (18.2)
	Don't know		8 (6.6)		8 (7.6)		11 (9.1)		25 (20.7)		1 (1.9)		3 (3.0)		5 (4.1)
Thrombotic thrombocytopenic purpura	Do not suspect at all	121	61 (50.4)	105	56 (53.3)	121	41 (33.9)	121	55 (45.5)	53	29 (54.7)	101	54 (53.5)	121	60 (49.6)
	Suspect		33 (27.3)		29 (27.6)		52 (43.0)		23 (19.0)		15 (28.3)		31 (30.7)		29 (24.0)
	Strongly suspect		18 (14.9)		13 (12.4)		16 (13.2)		18 (14.9)		8 (15.1)		13 (12.9)		27 (22.3)
	Don't know		9 (7.4)		7 (6.7)		12 (9.9)		25 (20.7)		1 (1.9)		3 (3.0)		5 (4.1)
Vasculitis	Do not suspect at all	121	42 (34.7)	105	30 (28.6)	121	23 (19.0)	121	10 (8.3)	53	11 (20.8)	101	21 (20.8)	121	21 (17.4)
	Suspect		51 (42.1)		50 (47.6)		53 (43.8)		60 (49.6)		26 (49.1)		48 (47.5)		52 (43.0)
	Strongly suspect		20 (16.5)		13 (12.4)		29 (24.0)		25 (20.7)		13 (24.5)		28 (27.7)		41 (33.9)

	Don't know		8 (6.6)		12 (11.4)		16 (13.2)		26 (21.5)		3 (5.7)		4 (4.0)		7 (5.8)
Aplastic anemia	Do not suspect at all	121	49 (40.5)	105	51 (48.6)	121	35 (28.9)	121	48 (39.7)	53	27 (50.9)	101	41 (40.6)	121	54 (44.6)
	Suspect		46 (38.0)		37 (35.2)		58 (47.9)		37 (30.6)		15 (28.3)		37 (36.6)		46 (38.0)
	Strongly suspect		14 (11.6)		6 (5.7)		10 (8.3)		9 (7.4)		8 (15.1)		18 (17.8)		15 (12.4)
	Don't know		12 (9.9)		11 (10.5)		18 (14.9)		27 (22.3)		3 (5.7)		5 (5.0)		6 (5.0)
Acquired coagulopathy	Do not suspect at all	121	23 (19.0)	105	14 (13.3)	121	16 (13.2)	121	12 (9.9)	53	7 (13.2)	101	10 (9.9)	121	16 (13.2)
	Suspect		41 (33.9)		27 (25.7)		56 (46.3)		39 (32.2)		19 (35.8)		31 (30.7)		50 (41.3)
	Strongly suspect		42 (34.7)		56 (53.3)		33 (27.3)		41 (33.9)		25 (47.2)		55 (54.5)		50 (41.3)
	Don't know		15 (12.4)		8 (7.6)		16 (13.2)		29 (24.0)		2 (3.8)		5 (5.0)		5 (4.1)
Henoch–Schönlein purpura (IgA vasculitis)	Do not suspect at all	121	64 (52.9)	105	34 (32.4)	121	24 (19.8)	121	19 (15.7)	53	12 (22.6)	101	35 (34.7)	121	26 (21.5)
	Suspect		39 (32.2)		38 (36.2)		53 (43.8)		51 (42.1)		28 (52.8)		40 (39.6)		63 (52.1)
	Strongly suspect		11 (9.1)		20 (19.0)		23 (19.0)		19 (15.7)		11 (20.8)		20 (19.8)		26 (21.5)
	Don't know		7 (5.8)		13 (12.4)		21 (17.4)		32 (26.4)		2 (3.8)		6 (5.9)		6 (5.0)
Subcutaneous hemorrhage due to trauma	Do not suspect at all	121	31 (25.6)	105	25 (23.8)	121	39 (32.2)	121	38 (31.4)	53	25 (47.2)	101	27 (26.7)	121	45 (37.2)
	Suspect		50 (41.3)		43 (41.0)		52 (43.0)		46 (38.0)		19 (35.8)		42 (41.6)		48 (39.7)
	Strongly suspect		33 (27.3)		31 (29.5)		22 (18.2)		11 (9.1)		8 (15.1)		26 (25.7)		24 (19.8)
	Don't know		7 (5.8)		6 (5.7)		8 (6.6)		26 (21.5)		1 (1.9)		6 (5.9)		4 (3.3)
Adverse drug	Do not suspect at all	121	29 (24.0)	105	22 (21.0)	121	22 (18.2)	121	22 (18.2)	53	18 (34.0)	101	23 (22.8)	121	29 (24.0)
	Suspect		51 (42.1)		39 (37.1)		60 (49.6)		50 (41.3)		18 (34.0)		43 (42.6)		55 (45.5)

reaction by anticoagulants	Strongly suspect		33 (27.3)		38 (36.2)		29 (24.0)		25 (20.7)		16 (30.2)		30 (29.7)		32 (26.4)
	Don't know		8 (6.6)		6 (5.7)		10 (8.3)		24 (19.8)		1 (1.9)		5 (5.0)		5 (4.1)
DIC	Do not suspect at all	121	31 (25.6)	105	49 (46.7)	121	39 (32.2)	121	44 (36.4)	53	27 (50.9)	101	34 (33.7)	121	49 (40.5)
	Suspect		45 (37.2)		39 (37.1)		47 (38.8)		41 (33.9)		14 (26.4)		40 (39.6)		40 (33.1)
	Strongly suspect		35 (28.9)		10 (9.5)		20 (16.5)		12 (9.9)		10 (18.9)		22 (21.8)		26 (21.5)
	Don't know		10 (8.3)		7 (6.7)		15 (12.4)		24 (19.8)		2 (3.8)		5 (5.0)		6 (5.0)
Acute leukemia	Do not suspect at all	121	34 (28.1)	105	47 (44.8)	121	26 (21.5)	121	32 (26.4)	53	15 (28.3)	101	32 (31.7)	121	38 (31.4)
	Suspect		46 (38.0)		42 (40.0)		48 (39.7)		43 (35.5)		20 (37.7)		38 (37.6)		45 (37.2)
	Strongly suspect		30 (24.8)		7 (6.7)		31 (25.6)		21 (17.4)		16 (30.2)		26 (25.7)		32 (26.4)
	Don't know		11 (9.1)		9 (8.6)		16 (13.2)		25 (20.7)		2 (3.8)		5 (5.0)		6 (5.0)
Other	Suspect	2	0 (0.0)	4	3 (75.0)	1	1 (100.0)	3	3 (100.0)	0	0 (-)	4	2 (50.0)	4	4 (100.0)
	Strongly suspect		2 (100.0)		1 (25.0)		0 (0.0)		0 (0.0)		0 (-)		2 (50.0)		0 (0.0)

AHA, acquired hemophilia A; DIC, disseminated intravascular coagulation; IgA, immunoglobin A.

Supplementary Table 3 continued: Diseases Suspected by Participants for the AHA Fictional Case After Receiving Laboratory Test Results (Stratified by Department).

Suspected disease	Level of suspicion	Urology		Gastroenterology surgery		Respiratory surgery		Neurosurgery		Rheumatology and collagen medicine		Geriatrics		Pediatrics		General internal medicine	
		n	n (%)	n	n (%)	n	n (%)	n	n (%)	n	n (%)	n	n (%)	n	n (%)	n	n (%)
Senile purpura	Do not suspect at all	121	66 (54.5)	121	63 (52.1)	105	54 (51.4)	121	67 (55.4)	110	88 (80.0)	6	2 (33.3)	121	67 (55.4)	253	156 (61.7)
	Suspect		19 (15.7)		38 (31.4)		35 (33.3)		25 (20.7)		15 (13.6)		1 (16.7)		35 (28.9)		52 (20.6)
	Strongly suspect		6 (5.0)		4 (3.3)		6 (5.7)		7 (5.8)		5 (4.5)		3 (50.0)		5 (4.1)		14 (5.5)
	Don't know		30 (24.8)		16 (13.2)		10 (9.5)		22 (18.2)		2 (1.8)		0 (0.0)		14 (11.6)		31 (12.3)
Idiopathic thrombocytopenic purpura	Do not suspect at all	121	55 (45.5)	121	53 (43.8)	105	50 (47.6)	121	57 (47.1)	110	76 (69.1)	6	2 (33.3)	121	85 (70.2)	253	144 (56.9)
	Suspect		30 (24.8)		35 (28.9)		34 (32.4)		28 (23.1)		24 (21.8)		3 (50.0)		26 (21.5)		59 (23.3)
	Strongly suspect		11 (9.1)		17 (14.0)		12 (11.4)		22 (18.2)		10 (9.1)		1 (16.7)		5 (4.1)		26 (10.3)
	Don't know		25 (20.7)		16 (13.2)		9 (8.6)		14 (11.6)		0 (0.0)		0 (0.0)		5 (4.1)		24 (9.5)
Thrombotic thrombocytopenic	Do not suspect at all	121	52 (43.0)	121	49 (40.5)	105	44 (41.9)	121	54 (44.6)	110	71 (64.5)	6	2 (33.3)	121	72 (59.5)	253	130 (51.4)
	Suspect		27 (22.3)		39 (32.2)		39 (37.1)		32 (26.4)		30 (27.3)		2 (33.3)		32 (26.4)		68 (26.9)
	Strongly suspect		16 (13.2)		17 (14.0)		13 (12.4)		23 (19.0)		8 (7.3)		2 (33.3)		11 (9.1)		32 (12.6)

purpura	Don't know		26 (21.5)		16 (13.2)		9 (8.6)		12 (9.9)		1 (0.9)		0 (0.0)		6 (5.0)		23 (9.1)
Vasculitis	Do not suspect at all	121	18 (14.9)	121	18 (14.9)	105	16 (15.2)	121	19 (15.7)	110	21 (19.1)	6	1 (16.7)	121	24 (19.8)	253	36 (14.2)
	Suspect		48 (39.7)		60 (49.6)		55 (52.4)		52 (43.0)		49 (44.5)		2 (33.3)		64 (52.9)		120 (47.4)
	Strongly suspect		27 (22.3)		26 (21.5)		24 (22.9)		28 (23.1)		38 (34.5)		2 (33.3)		26 (21.5)		71 (28.1)
	Don't know		28 (23.1)		17 (14.0)		10 (9.5)		22 (18.2)		2 (1.8)		1 (16.7)		7 (5.8)		26 (10.3)
Aplastic anemia	Do not suspect at all	121	46 (38.0)	121	44 (36.4)	105	43 (41.0)	121	53 (43.8)	110	67 (60.9)	6	3 (50.0)	121	60 (49.6)	253	119 (47.0)
	Suspect		36 (29.8)		54 (44.6)		44 (41.9)		41 (33.9)		33 (30.0)		2 (33.3)		45 (37.2)		82 (32.4)
	Strongly suspect		8 (6.6)		6 (5.0)		6 (5.7)		11 (9.1)		6 (5.5)		1 (16.7)		12 (9.9)		22 (8.7)
	Don't know		31 (25.6)		17 (14.0)		12 (11.4)		16 (13.2)		4 (3.6)		0 (0.0)		4 (3.3)		30 (11.9)
Acquired coagulopathy	Do not suspect at all	121	17 (14.0)	121	21 (17.4)	105	16 (15.2)	121	22 (18.2)	110	16 (14.5)	6	2 (33.3)	121	18 (14.9)	253	37 (14.6)
	Suspect		51 (42.1)		48 (39.7)		46 (43.8)		44 (36.4)		31 (28.2)		2 (33.3)		43 (35.5)		95 (37.5)
	Strongly suspect		30 (24.8)		38 (31.4)		33 (31.4)		37 (30.6)		61 (55.5)		2 (33.3)		56 (46.3)		87 (34.4)
	Don't know		23 (19.0)		14 (11.6)		10 (9.5)		18 (14.9)		2 (1.8)		0 (0.0)		4 (3.3)		34 (13.4)
Henoch–Schönlein purpura (IgA	Do not suspect at all	121	24 (19.8)	121	20 (16.5)	105	22 (21.0)	121	28 (23.1)	110	40 (36.4)	6	0 (0.0)	121	55 (45.5)	253	61 (24.1)
	Suspect		44 (36.4)		59 (48.8)		54 (51.4)		58 (47.9)		47 (42.7)		4 (66.7)		52 (43.0)		116

vasculitis)																	(45.8)
	Strongly suspect		22 (18.2)		23 (19.0)		16 (15.2)		18 (14.9)		23 (20.9)		1 (16.7)		8 (6.6)		49 (19.4)
	Don't know		31 (25.6)		19 (15.7)		13 (12.4)		17 (14.0)		0 (0.0)		1 (16.7)		6 (5.0)		27 (10.7)
Subcutaneous hemorrhage due to trauma	Do not suspect at all		48 (39.7)		39 (32.2)		36 (34.3)		45 (37.2)		46 (41.8)		3 (50.0)		43 (35.5)		103 (40.7)
	Suspect	121	33 (27.3)	121	49 (40.5)	105	43 (41.0)	121	39 (32.2)	110	43 (39.1)	6	0 (0.0)	121	54 (44.6)	253	90 (35.6)
	Strongly suspect		14 (11.6)		21 (17.4)		20 (19.0)		22 (18.2)		21 (19.1)		3 (50.0)		17 (14.0)		36 (14.2)
	Don't know		26 (21.5)		12 (9.9)		6 (5.7)		15 (12.4)		0 (0.0)		0 (0.0)		7 (5.8)		24 (9.5)
Adverse drug reaction by anticoagulants	Do not suspect at all		27 (22.3)		25 (20.7)		17 (16.2)		26 (21.5)		26 (23.6)		3 (50.0)		26 (21.5)		66 (26.1)
	Suspect	121	44 (36.4)	121	54 (44.6)	105	54 (51.4)	121	46 (38.0)	110	44 (40.0)	6	2 (33.3)	121	56 (46.3)	253	106 (41.9)
	Strongly suspect		25 (20.7)		28 (23.1)		28 (26.7)		36 (29.8)		39 (35.5)		1 (16.7)		37 (30.6)		56 (22.1)
	Don't know		25 (20.7)		14 (11.6)		6 (5.7)		13 (10.7)		1 (0.9)		0 (0.0)		2 (1.7)		25 (9.9)
DIC	Do not suspect at all		48 (39.7)		52 (43.0)		38 (36.2)		48 (39.7)		44 (40.0)		1 (16.7)		47 (38.8)		97 (38.3)
	Suspect	121	41 (33.9)	121	42 (34.7)	105	45 (42.9)	121	36 (29.8)	110	41 (37.3)	6	3 (50.0)	121	41 (33.9)	253	87 (34.4)
	Strongly suspect		12 (9.9)		14 (11.6)		13 (12.4)		25 (20.7)		24 (21.8)		2 (33.3)		27 (22.3)		41 (16.2)
	Don't know		20 (16.5)		13 (10.7)		9 (8.6)		12 (9.9)		1 (0.9)		0 (0.0)		6 (5.0)		28 (11.1)
Acute	Do not suspect at	121	30 (24.8)	121	19 (15.7)	105	28 (26.7)	121	38 (31.4)	110	47 (42.7)	6	2 (33.3)	121	41 (33.9)	253	71 (28.1)

leukemia	all																
	Suspect		46 (38.0)		54 (44.6)		45 (42.9)		47 (38.8)		47 (42.7)		3 (50.0)		56 (46.3)		89 (35.2)
	Strongly suspect		23 (19.0)		29 (24.0)		21 (20.0)		19 (15.7)		13 (11.8)		1 (16.7)		17 (14.0)		64 (25.3)
	Don't know		22 (18.2)		19 (15.7)		11 (10.5)		17 (14.0)		3 (2.7)		0 (0.0)		7 (5.8)		29 (11.5)
Other	Suspect	0	0 (-)	3	2 (66.7)	1	1 (100.0)	3	3 (100.0)	2	0 (0.0)	0	0 (-)	1	1 (100.0)	6	2 (33.3)
	Strongly suspect		0 (-)		1 (33.3)		0 (0.0)		0 (0.0)		2 (100.0)		0 (-)		0 (0.0)		4 (66.7)

AHA, acquired hemophilia A; DIC, disseminated intravascular coagulation; IgA, immunoglobin A.

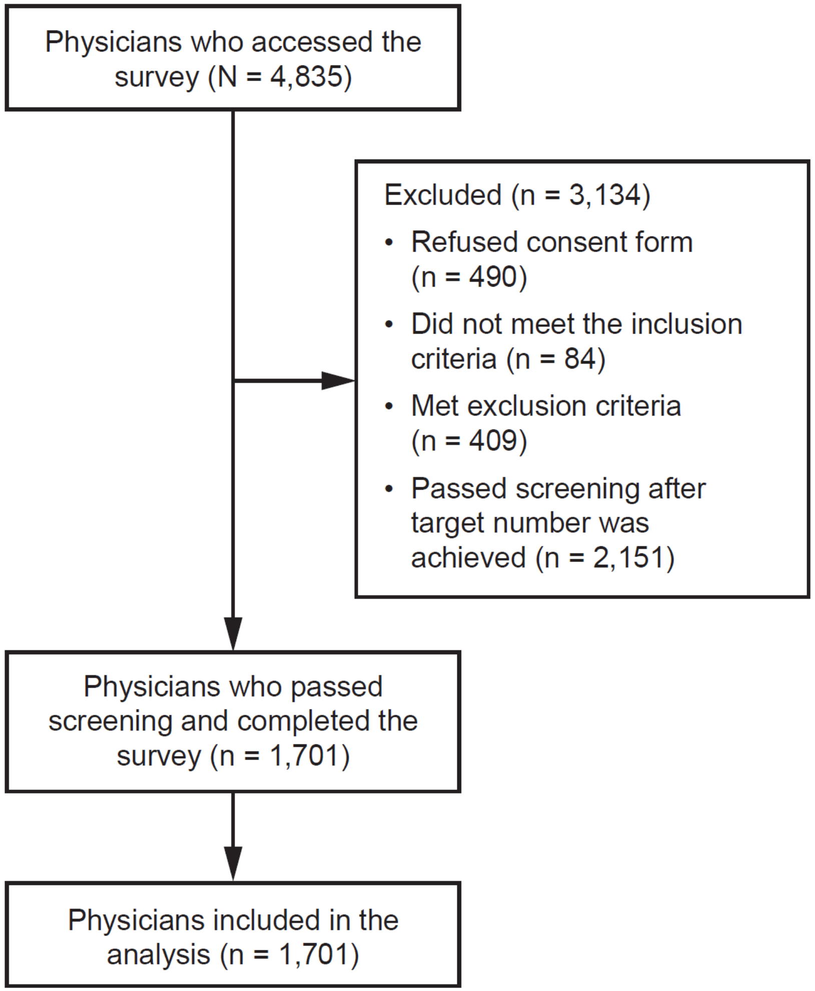

Supplementary Figure 1. Participant flow.
